# Supplementary material for: Virulence Factors and Antimicrobial Resistance Profile of Escherichia Coli Isolated from Laying Hens in Italy
Source: Animals (Basel). 2022 Jul 15;12(14):1812. doi: 10.3390/ani12141812 (PMC9311855; doi:10.3390/ani12141812)
Supplement: Supplementary file 1 [file animals-12-01812-s001.zip › animals-1805631-supplementary.pdf]

Table S1. The table reports the susceptibility of the 71 isolated *E. coli* strains to the considered antimicriobials. The results are expressed according to the breakpoint of CLSI and guidelines by CRAB.

**Antimicrobial drugs**	**Disk concentration**	**S[1]**		**I[2]**		**R[3]**	
		**Number**	**%**	**Number**	**%**	**Number**	**%**
nalidixic acid	30 µg	34	48	2	3	35	49
Aminosidine	60 µg	67	94	1	1	3	4
amoxicillin/clavulanic acid	10/20 µg	54	76	8	11	9	13
ampicillin	10 µg	40	56	4	6	27	38
apramycin	15 µg	70	99	0	0	1	1
cephalothin	30 µg	55	77	7	10	9	13
ceftiofur	30 µg	71	100	0	0	0	0
enrofloxacin	5 µg	68	96	2	3	1	1
florfenicol	30 µg	71	100	0	0	0	0
gentamicin	10 µg	68	96	0	0	3	4
kanamycin	30 µg	67	94	1	1	3	4
tetracycline	30 µg	53	75	1	1	17	24
sulfamethoxazole/trimethoprim	1.25/23.75 µg	65	92	0	0	4	6

[1]S = sensitive.

[2]I= increased exposure sensitivity.

[3]R=resistant.

Table S2. Antimicrobial-resistance profile of isolated *E. coli* strains, serogroups O2 and O88.

**Antimicrobial drugs**	**O2 Strains**			**O88 Strains**		
	**S[1]**	**I[2]**	**R[3]**	**S[1]**	**I[2]**	**R[3]**
nalidixic acid	2	0	13	9	0	0
aminosidine	14	1	0	8	0	1
amoxicillin/clavulanic acid	14	1	0	9	0	0
ampicillin	10	0	5	6	1	2
apramycin	15	0	0	9	0	0
cephalothin	13	1	1	8	1	0
ceftiofur	15	0	0	9	0	0
enrofloxacin	14	1	0	9	0	0
florfenicol	15	0	0	9	0	0
gentamicin	15	0	0	9	0	0
kanamycin	15	0	0	8	1	0
tetracycline	14	0	1	8	0	1
sulfamethoxazole/trimethoprim	13	0	1	9	0	0

[1]S = number of sensitive strains.

[2]I= number of increased exposure sensitivity strains.

[3]R= number of resistant strains.

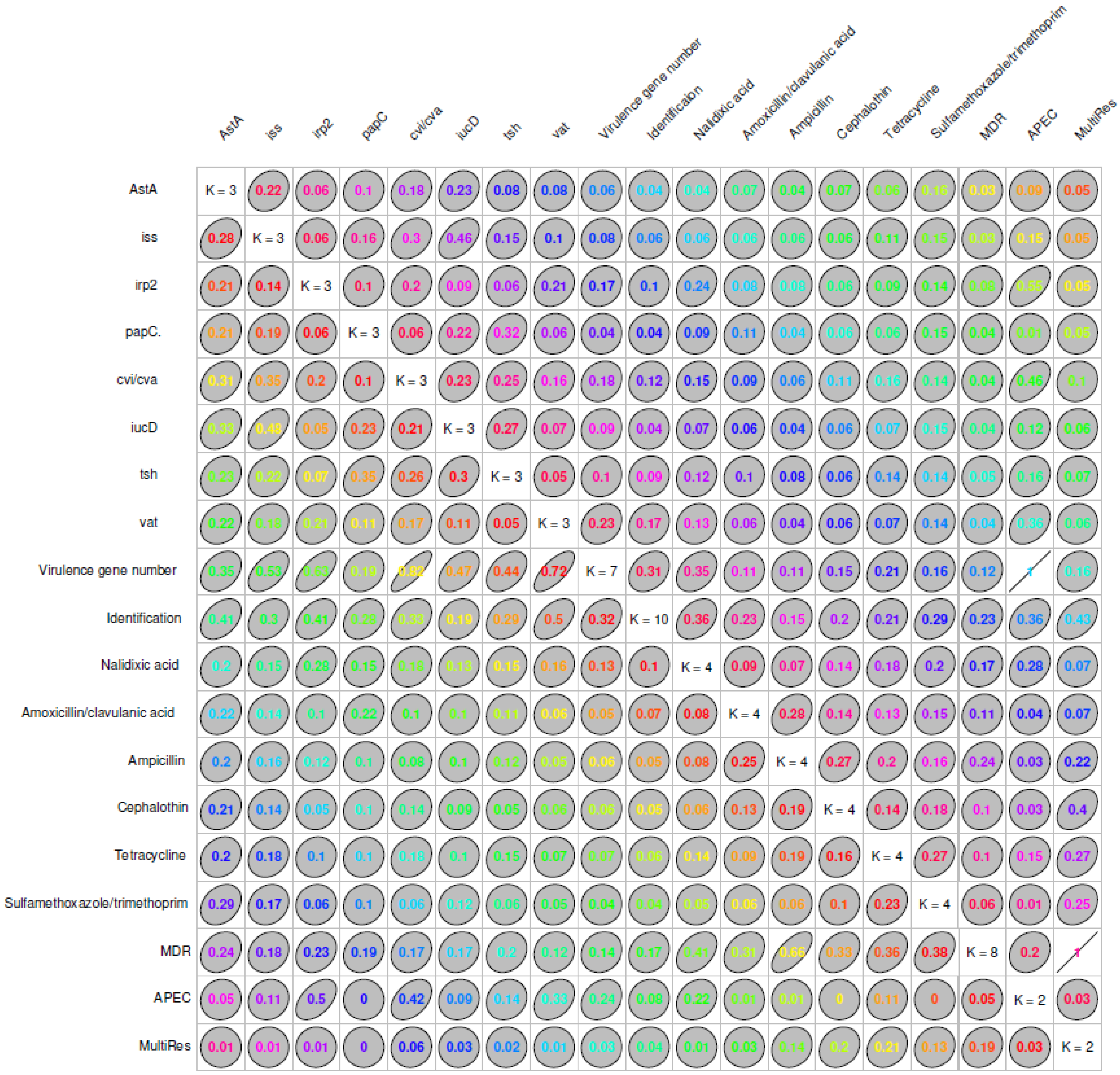

Figure S1. Statistical correlations between variables with Goodman – Kruskal tau.
